# Latin American Agricultural Workers' Job Demands and Resources and the Association With Health Behaviors at Work and Overall Health

**DOI:** 10.3389/fpubh.2022.838417

**Published:** 2022-04-07

**Authors:** Natalie V. Schwatka, Diana Jaramillo, Miranda Dally, Lyndsay Krisher, Lynn Dexter, Jaime Butler-Dawson, Rebecca Clancy, Gwenith G. Fisher, Lee S. Newman

**Affiliations:** ^1^Department of Environmental & Occupational Health, Center for Health, Work & Environment, Colorado School of Public Health, University of Colorado, Anschutz Medical Campus, Aurora, CO, United States; ^2^Department of Psychology, Colorado State University, Fort Collins, CO, United States; ^3^Department of Epidemiology, Colorado School of Public Health, University of Colorado, Anschutz Medical Campus, Aurora, CO, United States

**Keywords:** Total Worker Health, occupational health and safety, health promotion, worksite wellness, global health (MeSH [H02.403.371]), job demands, job resources

## Abstract

In the present study, we describe the job demands and job resources (JD-R) experienced by agricultural workers in three Latin American countries and their relationship to proactive health behaviors at work and overall health. Following previous research on the JD-R model, we hypothesized that job demands (H1) would be negatively related to agricultural workers' self-reported overall health. On the other hand, we hypothesized that job resources (H2) would be positively related to agricultural workers' overall health. Furthermore, we hypothesized (H3) that workers' engagement in jobsite health promotion practices via their proactive health behaviors at work would partially mediate the relationship between workers' job resources and job demands and overall health. We also had a research question (R1) about whether there were differences by type of job held. The sample of workers who participated in this study (*N* = 1,861) worked in Mexico, Guatemala, and Nicaragua for one large agribusiness that produces sugar cane. They worked in two distinct areas: company administration and agricultural operations. We administered employee health and safety culture surveys using survey methods tailored to meet the needs of both types of workers. Stratified path analysis models were used to test study hypotheses. In general, we found support for hypotheses 1 and 2. For example, operations workers reported more physically demanding jobs and administrative workers reported more work-related stress. Regardless, the existence of high job demands was associated with poorer overall health amongst both types of workers. We found that workers in more health-supportive work environments perform more proactive health behaviors at work, regardless of their role within the organization. However, hypothesis 3 was not supported as proactive health behaviors at work was not associated with overall health. We discuss future research needs in terms of evaluating these hypotheses amongst workers employed by small- and medium-sized agribusinesses as well as those in the informal economy in Latin America. We also discuss important implications for agribusinesses seeking to develop health promotion programs that meet the needs of all workers.

## Introduction

The Total Worker Health® (TWH) paradigm identifies and improves upon organizational approaches that contribute to workforce health, safety, and well-being (1). Historically, the field of occupational health and safety focused on the workplace hazards that resulted in work-related injury, illness and fatality. The TWH approach expands upon this to focus on the whole health of the worker, recognizing that working conditions can contribute to multiple dimensions of well-being (2). One way to conceptualize working conditions is with the Job Demands-Resources (JD-R) model, which categorizes working conditions into either job demands or job resources. The model has been applied to numerous contexts, including to understand and improve employee health and safety (3–5). In the present study, we aim to apply the JD-R model to understand how working conditions contribute to worker overall health and to extend this model to an underserved population of workers—Latin American agriculture workers.

Across all working contexts, the JD-R model indicates that two kinds of job characteristics are associated with employee engagement at work and with worker health and well-being. Job demands refer to “physical, psychological, social, or organizational aspects of the job that require sustained physical and/or psychological (cognitive and emotional) effort or skills” (p. 312) (3). Job demands are not inherently stressful or always perceived negatively. Job resources are typically perceived as positive as they motivate employees and lead to work engagement. Job resources are “(1) functional in achieving work goals, (2) reduce job demands and the associated physiological and psychological costs, and/or (3) stimulate personal growth, learning, and development” (p. 312) (3). Employees with high job demands but inadequate resources to meet those demands are at most risk for poor well-being. In this scenario, employees are more likely to perceive job demands as stressful and will be more likely to experience negative health outcomes. The JD-R model has typically been applied to aid in the understanding of burnout and work engagement (6), and is currently a popular and well-supported framework in occupational health psychology for investigating the associations between job characteristics and employee well-being (7). A recent meta-analysis utilized longitudinal evidence to validate the essential assumptions within the JD-R model, and the findings suggested the JD-R model is an excellent theoretical basis to assess employee well-being for a broad range of organizations (7). In the present study we aim to extend this model to understand behavior and overall health.

Several job demands can influence overall health. For example, exposure to hazardous working conditions can increase workers' risk for injury, illness, and death. The physical and psychological demands of work deplete workers' resources that may contribute to or support worker health. For example, factor such as poor relationships at work and time pressures can lead to chronic work-stress and result in maladaptive responses that can influence long-term health outcomes (8, 9). Additionally, workers who repeatedly perform physically demanding tasks are at risk for musculoskeletal disorders (10). Even just the presence of hazards can lead to perceptions that their work is dangerous to their health (11). Additionally, work design issues around work schedules and hours worked influence workforce health behaviors and health outcomes (12).

A supportive working environment is a critical job resource for health. Leaders should consistently support health initiatives at work both verbally and through demonstration (13). Organizations that develop an organizational health climate whereby employees perceive that their organization cares for and is committed to their health is another related, but distinct, job resource. Under these supportive conditions, employees may have better health (13). Finally, supervisors and co-workers who provide workers with advice and assistance facilitate connection and meaning at work that is related to well-being (14).

Job demands and resources influence worker health outcomes, in part, via their influence on work engagement (7). When employees have access to job resources, they will be more motivated to engage in health promoting activities at work. For example, research demonstrates that workers who perceive that their organization cares for and is committed to their health are more engaged in worksite health promoting activities, because they have more internal motivation to do so (15). On the other hand, exposure to job demands depletes workers' physical and psychological resources and decrease their motivation to engage in worksite health promoting activities. In other words, when workers are exposed to job demands they will be less likely to engage in activities that promote their health at work and thus prone to poorer health.

There is some research that applied the JD-R model to understanding engagement in workplace health promotion policies and programs and health. Job resources, such as perceived organizational support for wellness (16), are positively related to wellness program participation. On the other hand, job demands, such as long work hours, shiftwork, and working in more physically demanding occupations, are negatively associated with wellness program participation (17). Studies often find low participation rates in these health promotion initiatives, thus making an understanding of the JD-Rs associated with engagement in workplace health promotion policies and programs important (18). The JD-R model has also been used to understand health outcomes, such as depression and mortality (19, 20). Researchers apply this model to working populations around the globe, but mostly amongst workers in higher income countries like United Kingdom, Europe, Canada, and Australia (4). However, we are unaware of research that simultaneously considers the JD-R that are associated with engagement in workplace health promotion policies and programs and overall health amongst agricultural workers in the low- and middle-income countries (LMIC) of Latin America.

It is important to understand the health of agricultural workers in the Americas (21). Agricultural workers are exposed to a variety of workplace hazards, such as machinery and pesticides, that place them at risk for work-related injuries, illnesses, and fatalities. These exposures can vary depending on occupation. They can also vary by country due to differences in national regulations, employment conditions, benefits, etc. Globally, the agricultural sector is the third most hazardous industry to work in with almost twice the risk of sustaining a fatal injury as workers in other industries (22). The First Central American Survey of Working Conditions and Health in 2011 found that workers reported a significant number of concerns related to workplace safety, ergonomics, industrial hygiene and psychosocial hazards (23). The most recent data available from the International Labor Organization (ILO) provides estimates of the incidence of agricultural occupational non-fatal and fatal injuries in some Latin American countries (24). However, these statistics likely underestimate the true incidence of non-fatal and fatal occupational injuries (21). Agricultural workers also experience concerning health outcomes, such as communicable and non-communicable diseases (21), poor mental health (23), and musculoskeletal disorders (25). A recent multi-national survey of Latin American workers indicated that workers in manual occupations reported the highest prevalence of poor self-reported health (26). More recently, new and emerging health risks, such as climate change and the COVID-19 pandemic, are complicating efforts to protect and promote Latin American agricultural worker health (27, 28). An understanding of how to promote this working population's health is needed. The JD-R may aid in the understanding of how to accomplish this goal (29). While the JD-R model has been applied to Latin American agricultural workers who have migrated to work in other countries (30), it has not been applied to agricultural workers residing in Latin America.

In the present study, we describe the job demands and job resources experienced by agricultural workers employed by a large agribusiness in three Latin American countries and their relationship to health behaviors at work and overall health. Following previous research on the JD-R model, we hypothesized that job demands (H1) – *hazard perception, shift work, work hours, work stress, and physical demands* – would be negatively related to agricultural workers' self-reported overall health. On the other hand, we hypothesized that job resources (H2) – *leadership commitment to health, health climate, supervisor support, and social support* – would be positively related to agricultural workers' overall health. Furthermore, we hypothesized (H3) that workers' perceived engagement in jobsite health promotion practices via their proactive health behaviors at work would partially mediate the relationship between workers' job resources and job demands and overall health. We tested these hypotheses amongst two populations of workers within the agricultural workforce—administrative workers and operations workers. Within the agricultural sector there are differences in job tasks performed between both types of workers and thus differences in factors that contribute to their health outcomes. Our research question (R1) was whether there were differences in the JD-Rs associated with proactive health behaviors at work and overall health as well as whether proactive health behaviors at work played a mediating role in both working populations.

## Methods

### Sample

The sample of workers who participated in this study (*N* = 1,861) were employed by a large agribusiness in Central America that produced sugar cane with operations in Guatemala, Nicaragua, and Mexico. Workers at this agribusiness worked in two distinct areas across multiple departments. Administrative workers included supervisors, coordinators, and administrative assistants. Operations workers included field workers (i.e., seeders and sugarcane cutters), field nurse aides, drivers, mill workers, mechanics, system operators, among others. In addition to a robust safety program, the business had policies and programs to support their employee's overall health. Both types of workers had access to health and safety promoting programs such as routine health checkups, on-site clinics staffed by company physicians and nurses, vaccination campaigns, and safety inspections. Tobacco prevention and alcohol policies were in place for all workers. On the other hand, some health promoting policies and programs differed by type of worker. Administrative workers in the Guatemalan headquarters unlike other workers did not have access to an onsite clinic, instead these workers had their own private clinics. Administrative workers had access to exercise facilities and/or discounts at private gyms as well as physical activity campaigns. Some operations workers who cut sugarcane were provided with meals and snacks in the fields during the workday. Many of these field workers also had access to hydration, shade, rest breaks, and active observation of safety practices by supervisors, field nurse aides and physicians who provided education around prevention of injuries, heat stress, and other illnesses.

The agribusiness participated in a multifaceted assessment of TWH in their organization with researchers from the Center for Health, Work and Environment at the University of Colorado. As part of this project, a representative sample of workers were asked to complete an employee health and safety culture survey. Company staff emailed and texted all administrative staff a link to an online version of the survey (*n* = 368, 40% response rate). We administered an in-person survey to a 20% convenient sample of operational staff within each department (*n* = 1,493) excluding the largest department, “Guatemala agriculture.” Given the size and homogeneous nature of this population we concluded a convenience sample of 3% would be representative and feasible to collect. We administered the in-person survey to the operational staff via local interviewers who were trained to record responses into a portable tablet. This study was approved by the Colorado Multiple Institute Review Board. Consent was obtained from each participant following a detailed explanation about the purpose of the study.

### Measures

We based the employee health and safety culture survey on the survey used in the United States based Small+Safe+Well study (31). Briefly, researchers conducted the Small+Safe+Well study with a sample of small businesses in Colorado to address the culture of safety and health in the workplace. The health and culture survey has been described in detail previously, including its reliability and validity (15, 32–35). For the purposes of the present study, employee health and safety culture surveys were administered separately to administration and operations workers. The purpose of constructing separate surveys was to ensure that the survey items were understandable to the target audience. For the administrative staff, the survey was professionally translated into Spanish and contained 120 items. For the operations staff, we undertook additional steps to ensure we accommodated the broad range of education and literacy levels. Following translation into Spanish, the research team and representatives from the organization reviewed survey items and response options. All items were pilot tested prior to administration. The final 70 items contained visual analog scales instead of Likert scale response options. For this study, we only include survey items that were asked among both administrative and operations workers. All survey items and response options are presented in Table 1. Variables were measured either as a single item or as the average across a set of related items. For variables that were measured by averaging at least two items, the reliability of the measure was given by noting the Cronbach alpha.

**Table 1:** Survey measures.

**Variable**	**Survey question(s)**	**Response options**
**Job resources**		
Leadership commitment to health (33, 34) (α = 0.86)	Leaders consistently communicate the importance of worksite wellness Leaders are role models for prioritizing worksite wellness	1–5, strongly disagree to strongly agree
Health climate (36) (α = 0.82)	Operations worker: How committed is Pantaleon to employee health and well-being Administrative worker: My organization is committed to employee health and well-being	1–5, strongly disagree to strongly agree
	My organization provides me with opportunities and resources to be healthy	
	My organization encourages me to speak up about issues and priorities regarding employee health and well-being	
Supervisor support (37)	I can count on my supervisor/manager for support when I need it	1–5, strongly disagree to strongly agree
Social support (37) (α = 0.70)	I have the opportunity to develop close friendships in my job	1–5, strongly disagree to strongly agree
	My supervisor is concerned about my welfare and health	
**Job demands**		
Perception of hazard	How hazardous do you think your work environment is to your health? Examples of workplace hazards include: falls from height, exposure to electricity, highway driving, working with machinery, hit by a patient, lifting, etc.”	1–5, No danger to extremely dangerous
Work stress (38)	How often do you have feelings of stress because of your work?	1–5, Never to always
Hours per week	How many hours do you typically work each day? (answer with numbers only)	Text (number, Min: 0, Max: 18)
	On average (more or less) How many days do you normally work per week?	
Shift work	Do you do shift work (e.g., nights, swing shift)	1= Yes
		2= No
Physical demands	Created by researchers in collaboration with company health and safety personnel via coding of job position titles to reflect level of physical activity required to complete work tasks.	1 = low 2 = medium 3 = high
**Proactive health behaviors at work (**39**)**	You voluntarily carry out tasks or activities that help to improve the worksite wellness program.	1–4, Strongly disagree to Agree
**Overall health (**38**)**	How would you rate your overall health?	1–5, Bad to excellent

### Analysis

We computed participant demographic characteristics and scores on all variables of interest using Stata version 14.2. We summarized the findings by reporting the means and standard deviations or frequencies and percentages, as appropriate. Differences between administrative and operations workers were assessed via chi-square tests or *t*-tests, as appropriate.

We used a path analysis framework to examine our study hypotheses. Path analysis is a statistical method to understand the effects of a set of independent variables on outcomes via different causal pathways. Researchers can specify a pattern of relationships between the variables and use multiple linear regression equations to estimate the significance of the effects in tandem. In doing so, the researcher can estimate both the direct effects of independent variables on outcome variables as well as the indirect effects of independent variables on outcomes via specified mediators.

We tested stratified path models for the administrative workers and operations workers. All variables in Table 1 were included in the operations worker model. However, for the administrative worker model, the physical demands variable was excluded because all administrative workers reported being in the low category, and thus not enough variation in the variable was present to estimate the effect of physical demands on their study outcomes. We conducted the path analyses in Mplus 8 version 1.8.6 software (40).

First, the association between each of the job demands and job resources variables, proactive health behaviors at work and overall health were assessed (i.e., direct effects). Next, we evaluated whether there was evidence of full or partial mediation (i.e., indirect effects) via the Sobel test. We used the MODEL INDIRECT command and a bias-corrected bootstrapping method with 10,000 bootstrap samples to estimate the significance of the mediation effects (41). The total, direct, and indirect (i.e., mediation) effects were estimated. We controlled for age, gender, ethnicity, and country. Because the administrative and operations worker models were not nested, they could not be directly compared with model fit statistics.

## Results

Administrative and operations workers differed on all demographic indicators except for tenure with the company (see Table 2). Administrative workers were older and there were a greater proportion of females in the administrative sample than in the operations sample. Thirty four percent of the operations workers vs. only 7% of the administrative workers identified as Indigenous. The majority of administrative workers (94%) completed at least some college, but only 14% of the operations workers attended college. About two-thirds of the administrative workers worked in Guatemala (where the company headquarters is located) whereas there was a more even distribution of operations workers across all three countries.

**Table 2:** Description of study sample by type of worker (*N* = 1,861).

	**Administrative**		**Operations**	
	**Mean**	**SD**	**Mean**	**SD**
Age, years^***^	35	9	33	10
Tenure, years	9	9	9	9
	**Administrative**		**Operations**	
	***n***	**%**	***n***	**%**
Gender^***^				
Male	246	67%	1,260	85%
Female	119	33%	227	15%
Ethnicity^***^				
Mestizo	300	83%	644	43%
Indigenous	25	7%	513	34%
Other	38	10%	331	22%
Education^***^				
Less than high school	2	1%	956	64%
Completed high school	19	5%	321	22%
Completed at least some college/university	342	94%	213	14%
Schedule^***^				
All year	362	100%	883	60%
Harvest only	0	0%	591	40%
Country^***^				
Guatemala	232	63%	553	37%
Mexico	53	14%	220	15%
Nicaragua	82	22%	719	48%

*** *p < 0.001*,

**p < 0.01,

*p < 0.05 for a chi-square test or t-test, as appropriate.

As can be seen in Table 3, all of the job demands and resources variables were rated differently by type of worker. Operations workers rated leadership commitment to health, health climate, supervisor support, and social support significantly better than did administrative workers. Operations workers perceived that their job was more hazardous than did administrative workers. Administrative workers reported working fewer hours per week and had a smaller proportion of workers reporting that they engaged in shift work than operations workers. Operations workers reported engaging in more proactive health behaviors at work and reported better overall health than did administrative workers.

**Table 3:** Description of study variables by type of worker.

	**Administrative**		**Operations**	
	**M/N**	**SD/%**	**M/N**	**SD/%**
Leadership commitment to health^***^	4.10	0.83	4.44	0.54
Health climate^***^	4.29	0.77	4.45	0.53
Supervisor support^***^	4.36	0.78	4.52	0.59
Social support^***^	4.27	0.69	4.50	0.53
Hazard perception^***^	2.03	1.05	2.70	1.04
Work stress^***^	3.07	1.02	2.10	0.98
Hours worked per week^***^	46.70	18.96	56.58	14.12
Physical demands^***^				
Low	368	100%	249	17%
Medium	0%	0%	660	44%
High	0%	0%	580	40%
Shift work^***^				
Yes	60	17%	575	39%
No	300	82%	907	61%
Proactive health behaviors at work^*^	4.42	0.72	4.49	0.54
Overall health^***^	3.66	0.85	3.96	0.88

*** *p < 0.001*,

**p < 0.01,

* *p < 0.05*.

### Path Analysis Results

#### Administrative Workers

**Figure 1:**
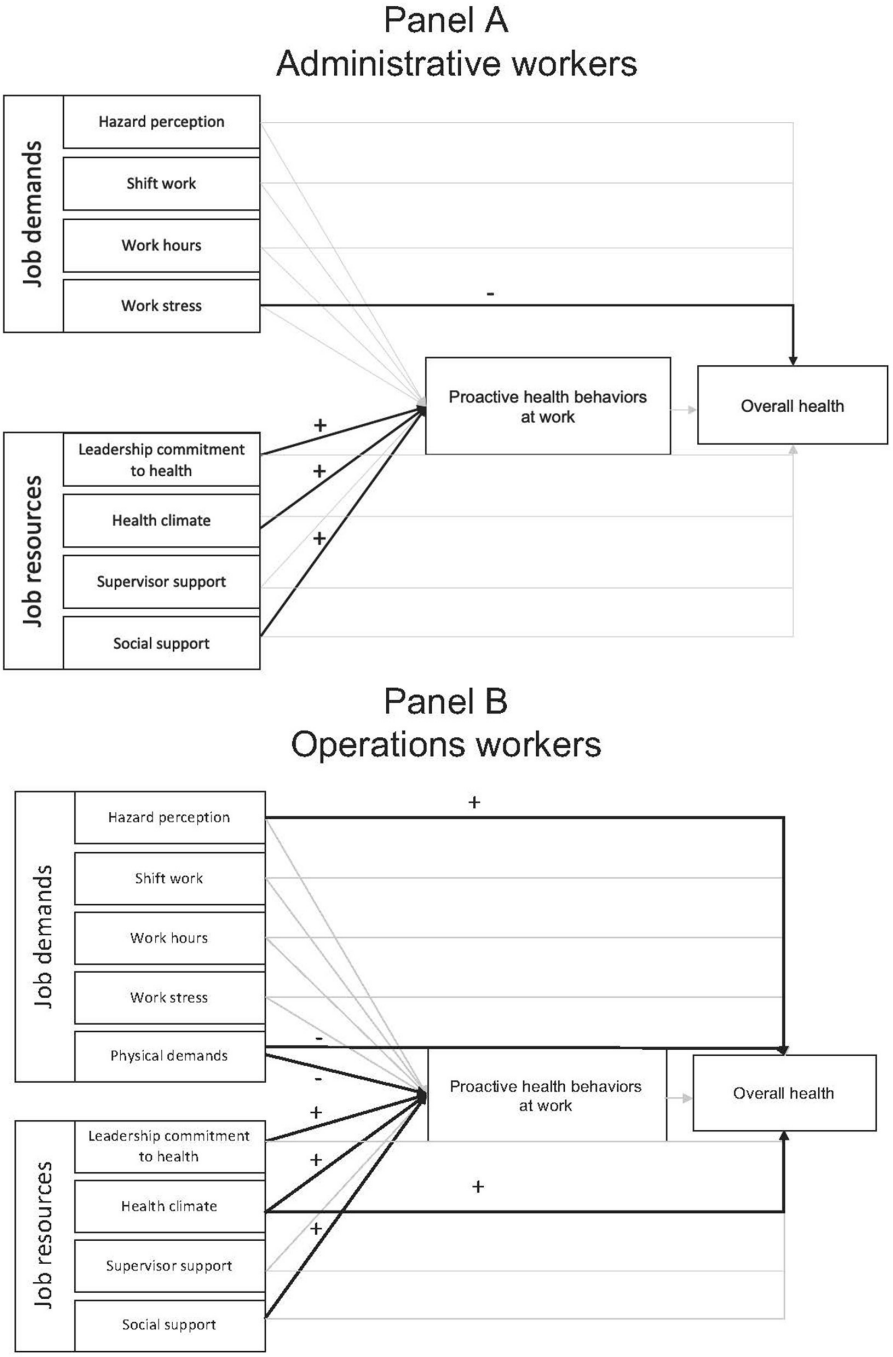
Final job demands-resources model of workplace health amongst administrative and operations agricultural workers in Latin America. Controlling for the effects of age, gender, country in which they worked, and ethnicity. Significant paths depicted by bold, black arrows. Insignificant paths depicted by grey arrows. The direction of the effect is depicted with either a “−” for a negative relationship or a “+” for a positive relationship.

We hypothesized that job demands (H1) – *hazard perception, shift work, work hours, work stress, and physical demands* – would be negatively related to agricultural workers' self-reported overall health. The only job demands variable that was significantly associated with overall health was stress at work where for one unit increase in work stress there was a 0.19 unit [SE (standard error) = 0.07] decline in perception of overall health. None of the job resource variables (H2) – *leadership commitment to health, health climate, supervisor support, and social support* – were significantly related to administrative workers' overall health. We hypothesized (H3) that workers' perceived engagement in jobsite health promotion practices via their proactive health behaviors at work would partially mediate the relationship between workers' job resources and job demands and overall health. None of the job demands variables were associated with administrative workers' proactive health behaviors at work. Of the job resource variables, leadership commitment to health, health climate, and social support were positively related to administrative workers' proactive health behaviors at work. For example, for a one unit increase in health climate there was a 0.24 unit (SE = 0.09) increase in proactive health behaviors at work. However, administrative workers' proactive health behaviors at work were not significantly related to their overall health and thus no evidence of mediation was detected. The final estimates for all significant direct effects can be found in Table 4 and are displayed in Panel A of Figure 1. The relationships proposed in the model explained 29% of the variance of administrative workers' proactive health behaviors at work and 16% of the variance of administrative workers' overall health.

**Table 4:** Path analysis results by type of worker.

	**Proactive health**		**Overall health**	
	**behaviors at work**			
	**β**	**SE**	**β**	**SE**
**Leadership commitment to health**				
Administrative worker	0.18^*^	0.08	0.04	0.07
Operations worker	0.26^***^	0.04	−0.04	0.04
**Health climate**				
Administrative worker	0.24^**^	0.09	0.10	0.08
Operations worker	0.25^***^	0.04	0.21^***^	0.05
**Supervisor support**				
Administrative worker	−0.07	0.07	−0.00	0.08
Operations worker	−0.00	0.03	−0.00	0.04
**Social support**				
Administrative worker	0.28^***^	0.09	0.12	0.09
Operations worker	0.11^**^	0.04	−0.01	0.04
**Hazard perception**				
Administrative worker	0.07	0.07	0.05	0.07
Operations worker	0.00	0.02	0.06^**^	0.03
**Work stress**				
Administrative worker	0.02	0.06	−0.19^**^	0.07
Operations worker	−0.03	0.02	−0.05	0.03
**Hours worked per week**				
Administrative worker	0.05	0.05	−0.05	0.05
Operations worker	−0.01	0.02	0.03	0.03
**Physical demands**				
Administrative worker	n/a	n/a	n/a	n/a
Operations worker	−0.07^**^	0.02	−0.07^*^	0.03
**Shift work**				
Administrative worker	0.04	0.05	−0.02	0.06
Operations worker	0.01	0.02	−0.04	0.03
**Proactive health behaviors at work**				
Administrative worker	n/a	n/a	0.09	0.07
Operations worker	n/a	n/a	0.05	0.04

*** *p < 0.001*,

** *p < 0.01*,

* *p < 0.05*.

#### Operations Workers

A few job demands and job resource variables had a significant relationship with operations workers' overall health. Regarding hypothesis 1, two job demands were significantly related to operations workers' overall health. Hazard perception was positively related to overall health whereas physical demands were negatively related to overall health. For example, for a one unit increase in physical demands there was a 0.07 unit (SE = 0.03) decline in operations workers' perception of their overall health. Regarding hypothesis 2, only health climate was positively related to operations workers' overall health where for a one unit increase in health climate there was a 0.21 unit (SE = 0.05) increase in operations workers' overall health. Finally, the hypothesis (H3) that workers' perceived engagement in jobsite health promotion practices via their proactive health behaviors at work would partially mediate the relationship between workers' job resources and job demands and overall health was not supported for operations workers. Of the job demands variables, operations workers' physical demands were negatively associated with their proactive health behaviors at work. For a one unit increase in physical demands there was a 0.06 unit (SE = 0.02) decline in proactive health behaviors at work. Of the job resource variables, leadership commitment to health, health climate, and social support were positively related to operations workers' proactive health behaviors at work. For example, for a one unit increase in health climate there was a 0.25 unit (SE = 0.04) increase in proactive health behaviors at work. Proactive health behaviors at work were not significantly related to overall health and thus no evidence of mediation was detected. The final estimates for all significant direct effects can be found in Table 4 and are displayed in Panel B of Figure 1. The relationships proposed in the model explained 52% of the variance of operations workers' proactive health behaviors at work and 13% of the variance of operations workers' overall health.

## Discussion

The present study draws upon the Job Demands Job Resources model to understand the factors associated with the perceived overall health of Latin American agriculture workers and to determine if differences existed between those who worked in administrative or operations roles. Our results suggest that there are commonalities and differences in both the job demands and job resources (1) that are experienced by both groups of workers and (2) that are associated with engagement in health promotion at work and overall health amongst both worker populations. We discuss our findings in detail below and offer suggestions for future research and practice to promote the health of Latin American agricultural workers.

### Job Resources

Our findings highlight important differences in perceptions of job resources within an organization. Specifically, we observed that operations workers rated job resources higher than did administrative workers. Prior research has also observed group differences in perceptions of job resources, such as between white and blue collar workers (42), supervisors and workers (43), and union and non-union workers (44), all with the former rating their job resources better than the latter. These results may be due, in part, to the theory of social comparison which states that people evaluate themselves and their circumstances based on comparisons with others (45). In the setting of Latin America, access to primary and occupational healthcare services is limited (46, 47). Upon hire at the organization in the study, operations workers (and in some cases, their families) are given access to clinical health services, health screening, health education, and in the case of some of the field workers, access to hydration, nutrition, and even healthcare and health promotion in the fields. This may position them to rate their perceptions of job resources higher. Another reason may be that health and safety efforts at the company are more visible to them and discussed more frequently with them, thus these resources may be more salient. For example, many operations workers participate in morning health and safety “momentos de dialogo,” which are akin to toolbox talks. Additionally, these workers receive education and assistance from nurses and physicians during work hours. The lack of visible and consistent attention to job resources for administrative workers may have contributed to their poorer perceptions of job resources. Additionally, administrative workers are more educated and thus may be more aware of the breadth and depth of what their organization could be doing for their health and thus more critical in their appraisals.

Regardless of these differences, we found that organizational supports can motivate both administrative and operations workers to engage in proactive health behaviors at work. These findings are consistent with literature demonstrating that job resources have a stronger relationship with job engagement than job demands (3). Indeed, prior health protection and promotion research also demonstrated that job resources are positively related to engagement in safety and health behaviors at work (11, 16, 48). Our study adds to this literature by demonstrating that supportive work environments are universally important for generating engagement in worksite health promotion initiatives across different working populations.

### Job Demands

The prevalence of job demands also differed by working population. More operations workers were exposed to physically demanding jobs than administrative workers. For administrative workers, a psychological job demand, work stress, was more common. Schreuder et al. (49) similarly found that blue collar workers reported more physical demands and white collar workers reported more psychological demands at work in a European working population. Administrative workers are likely performing more cognitively demanding work tasks, potentially with expectations that are difficult to meet, without enough recovery between tasks. Although we found administrative workers report working fewer hours than operations workers in our study, we surmise that they may have reported work hours spent physically in the office. Administrative workers likely work more hours outside of the typical workday at home and on the weekends. Relatedly, they are likely unable to “mentally checkout” of work after the workday ends, which can interfere with recovery from work and may experience more work/life balance challenges (50).

Amongst both types of jobs, workers who perceived their job to have higher demands were more likely to report worse overall health than workers who perceived their job to have lower demands. However, we observed role differences in the type of job demand that mattered. Amongst administrative workers, work stress was negatively related to their overall health. For operations workers, a physically demanding job was negatively associated with their overall health. Both physical and psychological job demands can negatively influence overall health, but they may target different aspects of health. We were not able to capture different aspects of health with our single item self-report perceptions of overall health. However, our findings demonstrate that we need to tailor interventions to reduce certain types of stressors based on the demands perceived within a role.

These factors could have also contributed to the differences we observed in hazard perceptions and ratings of their overall health between types of workers. Prior research has found that Latin American workers in manual occupations report worse health than workers in non-manual occupations (26). We did not observe the same finding in the present study. In fact, operations workers who reported a more hazardous work environment reported better overall health. Although operations workers are employed in physically demanding jobs that they perceive to be hazardous, they may perceive better health because of the access to health services that that would not have had access to otherwise. Another possible explanation for this finding is that workers in more physically demanding jobs may factor in the fact that they are physically able to do their demanding job. Additionally, operations workers were, on average, younger than administrative workers.

### Role of Proactive Health Behaviors at Work

Our hypothesis that the relationships between JD-Rs and overall health would be partially explained by workers' proactive health behaviors at work was not supported by our data. For example, we found evidence that health climate was associated with operations workers overall health. We did not observe the same relationship amongst administrative workers. We hypothesize that this may be due in part to a more established health climate amongst operations workers as discussed above. These findings are consistent with prior research demonstrating significant relationships between health climate and several health outcomes, including subjective general health, mental health, and work ability (51) as well as body mass index (52). However, this relationship could not be explained by their engagement in proactive health behaviors at work. It may be that JD-Rs are related to engagement in proactive health behaviors outside of work and this in turn is associated with overall health. Another alternative hypothesis that may help explain the pathway between JD-Rs is the effect of burnout (3); however, we were unable to measure the effect in the present study.

### Strengths and Limitations

The JD-R model has been frequently used in occupational health research to understand burnout and work engagement. A strength of the present study is the novel use of this model to understand other important outcomes: health behavior and overall health. Although our analysis could have been strengthened by including job burnout or work engagement in the model, we did not have access to this information. Another strength of the present study is the study population, which included workers from three Latin American LMICs. Even though the study population came from just one multinational company, there was good representation of workers from all departments and multiple countries. However, this is also a weakness of the study as it just represents the results from one, large company. Our results may have differed if we had we sampled agricultural workers from multiple small and medium sized organizations that often do not offer the same level of health promotion resources. Another limitation is the self-reported nature of the data and the uncertainty surrounding the reliability and validity of the survey measures. The survey was adapted from a United States-based worker survey that was found to be reliable and valid. However, to create a survey that was culturally relevant to and practical for this population we had to create two surveys and implement them via two formats to fit the needs of two distinct populations. The administration of the survey to operations workers via interview may have introduced a reporting bias as they may have felt the need to respond more favorably to questions than administrative workers who could complete their online survey on their own. Finally, the present study represents cross-sectional analyses and thus it was not possible to accurately test mediation and we cannot make any assumptions about causality.

### Future Research

The present study offers several avenues for future research in the JD-Rs that influence agricultural workers health. There is evidence that there may be gender differences in the experience of stress at work and its impact on health outcomes (53). The present study controlled for the effect of gender in an effort to understand the influence of occupation. However, future research should examine whether the hypothesized relationships differ by gender as well. The present study highlighted important within-business similarities and differences in JD-Rs and how they are related to overall health. In the future, it will be important to study whether these relationships hold in other Latin American agricultural businesses, especially small and medium sized companies that may not have similar levels of health promotion policies and programs. Perhaps more importantly, these relationships should be investigated in the large informal economy in Latin America (54). In doing so, it will be important to understand whether JD-Rs interact in their effect on work engagement and overall health. For example, does leadership commitment to health buffer the negative effect of physical job demands on work engagement and overall health amongst operations workers? It will also be useful to distinguish between challenging job demands that promote engagement and hindering job demands that detract from engagement (55). For example, what role does job responsibility, a challenging job demand, play in workers' proactive health behaviors at work? Workers lifestyle health risk factors and/or chronic health conditions may also play a role in the relationship between the physical demands of the job and their perceptions of their overall health (56). Relatedly, future studies should evaluate whether JD-Rs are related to specific health outcomes that are measured objectively. Finally, researchers should test interventions for each group of workers to determine if they can reduce job demands and enhance job resources to improve overall health.

### Practical Implications

The present study offers important implications for Latin American agribusinesses seeking to develop health promoting programs. First, employers should focus on creating an organizational climate that supports employee health. One way they can do this is through developing the leadership skills of formal leaders to support the use of health promoting policies and programs during and outside of work (13). In doing so, they will demonstrate to all workers that their health is valued. Our study suggests that when employers do this, they may boost workforce engagement in their worksite health promotion practices. Second, when developing specific health policies and programs, employers should consider needs of all workers. For example, in the present study's working population, a program aimed at stress reduction would predominately meet the needs of administrative workers, not operations workers. Finally, considering new and emerging risks, such as climate change and the COVID-19 pandemic, employers should apply the Total Worker Health approach when developing and implementing health promoting programs in order to consider multiple factors that may impact health and a broad definition of worker health and well-being (1, 57).

## Data Availability Statement

The datasets presented in this article are not readily available because of a data sharing agreement between Pantaleon and the University of Colorado under a Memorandum of Understanding. Portions of this data set are the property of Pantaleon, a third party. However requests to access available datasets will be considered without undue reservations. Requests to access the datasets should be directed to the corresponding author, NS. Requests to access the datasets should be directed to natalie.schwatka@cuanschutz.edu.

## Ethics Statement

The studies involving human participants were reviewed and approved by Colorado Multiple Institutional Review Board. The patients/participants provided their written informed consent to participate in this study.

## Author Contributions

NS made substantial contributions to the conception, design of the work, analysis, interpretation of the data, and drafting and revision of the work. DJ, MD, LK, JB-D, and RC made substantial contributions to the acquisition, analysis, interpretation of the data, and drafting and revision of the work. GF and LN made substantial contributions to the acquisition and interpretation of the data and drafting and revision of the work. All authors approved the submitted version and agreed to be personally accountable for the author's own contribution.

## Funding

This study was supported in part by Pantaleon, the Chancellor, CU Anschutz, and Centers for Disease Control and Prevention (CDC) U19OH01127, T42OH009229, and K01OH011726.

## Author Disclaimer

The contents are solely the responsibility of the authors and do not necessarily represent the official views of the U.S. Centers for Disease Control and Prevention or the Department of Health and Human Services.

## Conflict of Interest

The authors declare that the research was conducted in the absence of any commercial or financial relationships that could be construed as a potential conflict of interest.

## Publisher's Note

All claims expressed in this article are solely those of the authors and do not necessarily represent those of their affiliated organizations, or those of the publisher, the editors and the reviewers. Any product that may be evaluated in this article, or claim that may be made by its manufacturer, is not guaranteed or endorsed by the publisher.
